# Lipolysis and antioxidant properties of cow and buffalo cheddar cheese in accelerated ripening

**DOI:** 10.1186/s12944-018-0871-9

**Published:** 2018-10-02

**Authors:** Maryam Batool, Muhammad Nadeem, Muhammad Imran, Imran Taj Khan, Jalees Ahmad Bhatti, Muhammad Ayaz

**Affiliations:** 1 Department of Dairy Technology, University of Veterinary and Animal Sciences, Lahore, Pakistan; 2 Institute of Home and Food Sciences, Faculty of Life Sciences, Government College University, Faisalabad, Pakistan; 3 Department of Livestock Production, University of Veterinary and Animal Sciences, Lahore, Pakistan

**Keywords:** Buffalo milk, Cheddar cheese, Lipolysis, Fatty acid profile

## Abstract

**Background:**

Buffalo milk is the second largest source of milk on the globe, it is highly suitable for the preparation of mozzarella cheese, however, it is not suitable for the preparation of cheddar cheese due to high buffering capacity, low acid development, excessive syneresis, lower lipolysis that lead to lower sensory score. Accelerated ripening can enhance lipolysis and improve sensory characteristics of cheddar cheese. Lipolysis and antioxidant capacity of buffalo cheddar cheese in conventional ripening is not previously studied. Optimization of ripening conditions can lead to better utilization of buffalo milk in cheese industry.

**Methods:**

Effect of accelerated ripening on lipolysis and antioxidant properties of cow and buffalo cheddar cheese were investigated. Cheddar cheese prepared from standardized (3.5% fat) cow and buffalo milk was subjected to conventional and accelerated ripening (4 °C and 12 °C) for a period of 120 days. Fatty acid profile, organic acids, free fatty acids, cholesterol, antioxidant activity and sensory characteristics were studied at 0, 40, 80 and 120 days of ripening.

**Results:**

Fatty acid profile of cow and buffalo cheddar in conventional (120 days old) and accelerated ripening were different from each other (*p* < 0.05). Free fatty acids in 120 days old buffalo and control cheddar, in accelerated ripening were 0.55% and 0.62%. After accelerated ripening, cholesterol in buffalo and control cheddars were 16 and 72 mg/100 g. After accelerated ripening, concentrations of formic, pyruvic, lactic, acetic and citric acids in buffalo cheddar cheese were, 922, 136, 19,200, 468 and 2845 ppm. At the end of accelerated ripening (120 days), concentrations of formic, pyruvic, lactic, acetic and citric acids in cow cheddar cheese were 578, 95, 9600, 347 and 1015 ppm. Total antioxidant capacity of control cow and buffalo cheddar in accelerated ripening was 77.26 and 88.30%. Colour, flavour and texture score of rapid ripened 80 and 120 days old buffalo cheddar was not different from cow cheddar.

**Conclusions:**

Results of this investigations showed that flavour profile buffalo cheddar subjected to accelerate ripening was similar to cow cheddar cheese. Accelerated ripening can be used for better utilization of buffalo milk in cheddar cheese industry.

## Background

Buffalo milk contributes 12% of the total milk production in the world [1]. About 80% of total buffalo milk is produced in India and Pakistan [2]. Chemical composition of buffalo milk in terms of major and minor constituents is significantly different from cow milk. Buffalo milk has higher fat, protein, minerals and total solid contents than cow milk [3]. Concentration of cholesterol in buffalo milk ranged from 8 to 10 mg/100 g while, concentration of cholesterol in cow milk ranged from 30 to 35 mg/100 g [4]. Viscosity and calcium content of buffalo milk is also greater than cow milk [5]. As compared to cow milk, buffalo milk has higher levels of vitamin E, C, zinc, selenium and sulphur containing amino acids, these biochemical compounds are responsible for antioxidant activity in milk and milk products [6, 7]. Average size of fat globules in buffalo and cow milk is 8.7 μm and 0.1-5 μm, respectively [8]. Size of milk fat globule has a pronounced effect on manufacturing and ripening of cheese. Camembert cheese prepared from milk with large sized fat globules underwent lower degree of lipolysis [9]. Chemical composition of buffalo milk offers excellent opportunities for the production of various value-added dairy products; however, larger size of fat globules, higher buffering capacity, slower acid development and excessive syneresis makes buffalo milk unfit for making superb quality cheddar cheese. Results of earlier investigations showed that cheddar cheese prepared from buffalo milk underwent excessive syneresis, required long ripening time with lower textural and flavour prospects [10]. Cheddar is recognized as hard cheese and its acceptability mainly depends upon the flavour, which is produced during ripening [11]. Biochemical and metabolic processes lead to the significant changes in flavour and texture of cheese; these processes are glycolysis, lipolysis and proteolysis [12]. Lipolysis plays an important role in the breakdown of triglycerides of milk fat, generation of free fatty acids, organic acids, large number of flavouring compounds such as, thio-esters, alkan-2-ones, alkane-2-ols, ethyl alcohols, lactones etc. [13]. Ripening temperature has great effect on biochemical events which take place during the maturation of cheese, ripening of cheese at relatively higher temperatures can significantly accelerate the metabolic processes in cheese. Ripening of cheddar cheese is mandatory for the development of typical flavour and textural characteristics. Cheddar cheese is usually ripened for 8–12 months at 4-6 °C. Ripening of cheese on such a lower temperature for a fairly long period of time requires massive refrigeration and inventory cost. Accelerated ripening enhanced the concentration of organic acids in cheddar cheese [14]. However, in this study, fatty acid profile, free fatty acids, peroxide value, cholesterol and change in antioxidant properties of buffalo cheddar cheese in accelerated ripening was not investigated. Accelerated ripening may be used to improve the chemical and sensory prospects of buffalo feta cheese [15]. In view of the economic and nutritional importance of buffalo milk and its lack of suitability for the making of hard/ semi-hard cheese, new technologies should be tried or existing techniques should be explored to enhance the suitability of buffalo milk for the manufacturing of cheddar cheese. To our knowledge, fatty acid profile, organic acids, antioxidant and sensory properties of buffalo cheddar Cheese have not been previously investigated. This study aimed to determine the influence of accelerated ripening on lipolysis, antioxidant and sensory characteristics of buffalo cheddar cheese.

## Methods

### Materials

Cow and buffalo milk were obtained from Dairy Animals Training and Research Centre, University of Veterinary and Animal Sciences Lahore. Starter culture, *Streptococcus lactis* ssp*. lactis* and *Streptococcus lactis* ssp*. cremoris* were obtained from Christian Hansen, Denmark. Chemicals used in this investigation were HPLC grade and purchased from Sigma Aldrich, St. Louis, MO, USA.

### Experimental plan

Cheddar cheese was prepared from standardized buffalo and cow milk (3.5% fat). Cheddar cheese was ripened at 4 °C and 12 °C for a period of 120 days, analysed at 0, 40, 80 and 120 days of ripening period.

### Compositional analysis

Fat, protein and moisture content in cheese were determined by following the standard methods [16].

### Lipolysis

Fatty acid profile was determined by converting the fat into fatty acid methyl esters, briefly, 50 mg fat was taken in a 15 ml test tube, dissolved in 3-ml iso-octane and 2 ml 0.5 N sodium methoxide was added. Test tube was capped and vortex for 3-min at 2200 rpm, after 5 min, supernatant was transferred to GC vials and injected to GC-MS (79890-A Agilent, USA) fitted with fused silica capillary column (SP 2560; 100 m, film thickness 25 μm) and Mass Selective Detector [17]. Helium was used as carrier gas at the flow rate of 2 ml/ min; total run time was 35 min. Fatty acid standards, FAME 37 kit (Sigma Aldrich, UK) was used for identification and quantification of fatty acids. Cholesterol in cheese was determined according to the method [18]. Free fatty acids were determined in terms of oleic acid, for the determination of free fatty acids, absolute ethanol was neutralized to light pink colour, using 3 drops of 1% phenolphthalein indicator, 5 g fat was taken in the same flask, contents of flask were mixed, boiled for 2 min and titrated against standardized 0.1 N NaOH till light pink colour end point and free fatty acids (FFA) were calculated by the following formula [19].

$$ \%\mathrm{FFA}\;\left(\mathrm{Oleic}\ \mathrm{Acid}\right)=\frac{\mathrm{Volume}\kern0.17em \mathrm{of}\;0.1\;\mathrm{N}\;\mathrm{N}\mathrm{aOH}\;\mathrm{x}\;\mathrm{N}\mathrm{ormality}\kern0.17em \mathrm{of}\kern0.17em \mathrm{NaOH}\;\mathrm{x}\;282\;\mathrm{x}\;100}{1000\;\mathrm{x}\;\mathrm{Weight}\kern0.17em \mathrm{of}\kern0.17em \mathrm{Sample}} $$

### Peroxide value and iodine value

Peroxide value and iodine value were determined by following the standard methods [19].

### HPLC characterization of organic acids

Samples were prepared by adding 0.1 N phosphoric acid to 5 g grated cheese; organic acids were extracted by dispersing in homogenizer (Heidolph Diax 900, Schwabach, Germany) at 1500 rpm for 5 mins, followed by centrifugation at 7000 rpm for 15 min and supernatant was filtered. Organic acids were determined on HPLC (LCM, Waters Corp., Milford, MA, USA) fitted with ion exchange chromatography column (Supelcogel C-610H, 300 × 7.8 mm, Supelco Inc., Bellefonte, PA, USA). The mobile phase was comprised HPLC grade phosphoric acid with a flow rate of 1 ml/minute, injection volume was 40 μl and measurements were performed at 210 nm. Standards of citric, lactic, formic acids (99% pure, Sigma Chemical Company, St Louis, MO, USA) were used for the identification and quantification of organic acids on a calibration curve and reported as mg/kg dry matter [20].

### Antioxidant properties of water-soluble extracts

#### Preparation of water soluble extracts of Cheddar cheese

Water soluble extracts of cow and buffalo cheddar cheese were prepared according to the method prescribed by [21]. Sixty ml double distilled water was mixed with 20 g grated cheese and homogenized (Ultrasonicator, SONICS, VC 750, USA), homogenized stuff was centrifuged at 14000 g for 10 min (Heraeus, Centrifuge, Hanau, Germany). Supernatant fat layer was castoff, extracts were filtered by Whatman No.1 filter paper. For the precipitation of proteins, pH of the extracts was adjusted to 4.1 using 1 N HCl, contents were again filtered by Whatman No.1. Water-soluble extracts were lyophilized in a rotary evaporator (Buchi, Japan).

#### Total antioxidant activity

Total antioxidant activity in cheese samples was determined according to the method of [22]. Sample 1 ml was mixed with 3 ml each of 0.6 M sulphuric acid, 28 mM sodium phosphate and 4 mM ammonium molybdate). Test tubes were incubated at 85 °C for 90 min, absorbance was recorded at 695 nm in visible region of spectrum using reagent blank and ascorbic acid as standard. Total antioxidant activity was determined at 0, 40, 80 and 120 days of accelerated ripening and reported as mg Ascorbic Acid Equivalent per gram.

#### DPPH free radical scavenging activity

DPPH free radical scavenging activity of cow and buffalo cheddar cheese was determined by following the method of [23]. 1 ml sample was mixed with 1 ml DPPH solution (1 mM in methanol) and incubated at room temperature for 30 min. The absorbance was measured on a double beam spectrophotometer (Shimadzu, Japan). DPPH free radical scavenging activity was determined at 0, 40, 80 and 120 days of ripening and expressed as %inhibition.

#### Sensory evaluation

Sensory evaluation of cheese was performed by a panel of ten trained judges; cheese samples were tempered at 15 °C for 2 h, prior to evaluation. Samples were randomly coded with three-digit random numbers and offered for sensory evaluation in a well illuminated sensory evaluation laboratory at 20 °C, Samples were evaluated for colour, flavour and texture, on 9-point scale at 40, 80 and 120 days of ripening [24].

#### Statistical analysis

Experiment was planned in a completely randomized design; each treatment was replicated three times. Each sample was analysed three times, results were reported as Mean ± SE, and two-way analysis of variance technique was used to determine the effect of treatments, ripening temperatures and their interaction. T Test was used to determine the significant difference among the treatments (*p* < 0.05) on SAS 9.1 statistical software [25].

## Results and discussion

### Chemical composition

The results of chemical composition of cow and buffalo cheddar cheese are given in Table 1. Fat and protein content in buffalo cheddar cheese were higher than cow cheddar (*p* < 0.05). Higher protein content in buffalo cheddar was due to the higher concentration of casein in buffalo milk. Casein has a great effect on composition and yield of cheddar cheese [2]. Moisture content of buffalo cheddar was lower than cow cheddar (*p* < 0.05). The lower moisture content in buffalo cheddar cheese was due to the higher fat and protein in buffalo cheddar. Fat and protein content of cow and buffalo cheddar were significantly influenced by ripening temperature; after 120 days of conventional ripening, fat content of buffalo cheddar decreased from 32.79 to 31.27% (*p* < 0.05) while fat content of cow cheddar decreased from 30.55 to 29.25% (*p* < 0.05). After 120 days of conventional ripening, protein content of cow cheddar decreased from 24.83 to 23.28% while buffalo cheddar decreased from 27.18 to 26.14%. After 120 days of accelerated ripening, fat content of buffalo cheddar decreased from 32.79 to 29.23% while cow cheddar decreased from 30.51 to 26.85%. After 120 days of accelerated ripening, protein content of cow cheddar decreased from 24.87 to 22.27% while protein content in buffalo cheddar decreased from 27.18 to 25.36%. During cheese ripening, several biochemical and microbiological events take place, which lead to the breakdown of lipids, protein and carbohydrates [26]. Moisture, protein, fat and ash content in cheddar cheese were 37%, 25%, 33% and 4% [27]. Fat accomplishes numerous roles in cheese, it enhances taste, appearance, textural characteristics of cheese by filling intracellular spaces among protein and mineral matrix of cheese. Cheddar cheese prepared from buffalo milk had higher fat and protein content than cow cheddar [2]. Fat and protein content of cheddar cheese derived from the low melting fractions of milk fat decreased, during the ripening period of 90 days [28]. Fat, protein and moisture content of Gouda cheese decreased during the ripening process [29].

**Table 1:** Chemical composition of cow and buffalo cheddar cheese ripened at 4 °C and 12 °C

Ripening Temperature	Ripening Days	Cow Cheddar	Buffalo Cheddar	Cow Cheddar	Buffalo Cheddar	Cow Cheddar	Buffalo Cheddar
		Moisture %	Moisture %	Fat %	Fat %	Protein %	Protein %
4 °C	0	38.27 ± 1.15^a^	35.89 ± 1.28^b^	30.55 ± 0.84^b^	32.79 ± 0.93^a^	24.83 ± 0.55^d^	27.18 ± 0.61^a^
	40	38.21 ± 0.99^a^	35.62 ± 0.76^b^	30.12 ± 0.65^b^	32.55 ± 0.65^a^	24.64 ± 0.17^d^	27.05 ± 0.46^a^
	80	37.92 ± 0.42^a^	35.13 ± 0.48^b^	29.41 ± 0.27^c^	31.83 ± 0.17^b^	24.33 ± 0.36^d^	26.88 ± 0.32^b^
	120	37.73 ± 0.66^a^	34.26 ± 0.95^c^	29.25 ± 0.52^c^	31.27 ± 0.54^b^	23.28 ± 0.43^e^	26.14 ± 0.29^b^
12 °C	0	38.27 ± 1.15^a^	35.89 ± 1.28^b^	30.51 ± 0.84^b^	32.79 ± 0.93^a^	24.87 ± 0.55^d^	27.18 ± 0.61^a^
	40	38.12 ± 0.75^a^	35.42 ± 0.73^b^	29.33 ± 1.12^c^	32.18 ± 0.72^a^	24.54 ± 0.34^d^	27.11 ± 0.53^a^
	80	37.53 ± 0.46^a^	34.78 ± 1.05^c^	28.18 ± 0.39^d^	31.11 ± 0.19^b^	23.53 ± 0.49^e^	26.29 ± 0.98^b^
	120	36.19 ± 0.82^b^	33.19 ± 0.59^d^	26.85 ± 0.77^e^	29.23 ± 0.42^c^	22.27 ± 0.19^f^	25.36 ± 0.62^c^

Within the columns of a parameter, means denoted by different letter are statistically different (*p* < 0.05)

### Total antioxidant capacity (TAC)

Results of TAC of cow and buffalo cheddar cheese in conventional and accelerated ripening are given in Table 2. TAC of fresh and ripened buffalo cheddar was greater than cow cheddar and all the determinations revealed the same trend. In conventional and accelerated ripening, antioxidant activity of cow and buffalo cheddar cheese increased throughout the ripening period of 120 days. After 120 days of conventional ripening, TAC of cow and buffalo cheddar was 53.42 and 73.91%. Total antioxidant activity of 120 days old rapid ripened cow and buffalo cheddar cheese was 77.76% and 88.30%. Higher TAC of buffalo cheddar may be attributed to the higher concentration of sulphur containing amino acids, vitamin E, catalase, glutathione peroxidase activities, however, detailed investigation is required regarding the biochemical characterization of buffalo cheddar cheese in accelerated ripening. Gupta et al. [30] studied the antioxidant properties of cheddar cheese in conventional ripening at different stages of storage, TAC of cheese increased to 70% in four months, in current investigation, TAC of buffalo cheddar cheese after 80 days of ripening was 51.66%. TAC of 120 days old cow and buffalo cheddar in accelerated ripening was 77.26 and 88.30%. TAC of probiotic cheese increased with the progression of ripening period [31]. Kudoh et al. [32] Identified an antioxidant peptide in fermented milk. During the ripening of cheese, proteolysis lead to the formation of various peptides, these peptides not only contribute in the development of flavour and texture but also reveal a considerable functional value [33]. These antioxidant peptides present in cheese play a vivacious role to prevent the body from oxidative stress, which have been implicated in cancer, ageing, atherosclerosis and diabetes.

**Table 2:** Antioxidant properties of cow and buffalo milk cheddar cheese in accelerated ripening

Ripening Temperature	Ripening Days	Cow Cheddar	Buffalo Cheddar	Cow Cheddar	Buffalo Cheddar
		TAC	TAC	DPPH	DPPH
		%	%	%	%
4 °C	0	15.42 ± 0.61^l^	20.55 ± 1.46^k^	9.61 ± 0.62^l^	13.82 ± 1.24^k^
	40	27.36 ± 1.13^j^	30.72 ± 1.71^i^	19.37 ± 0.52^j^	29.88 ± 1.97i
	80	40.87 ± 1.59^g^	51.66 ± 2.19^f^	31.67 ± 1.16^h^	48.22 ± 1.73^e^
	120	53.42 ± 1.38^e^	73.91 ± 2.34^c^	44.56 ± 1.66^f^	63.27 ± 2.81^c^
12 °C	0	15.42 ± 0.88^k^	20.55 ± 0.92^k^	9.61 ± 0.75^l^	13.82 ± 0.62^k^
	40	32.91 ± 1.33^h^	37.43 ± 1.55^h^	23.46 ± 1.75^i^	39.88 ± 2.11^g^
	80	65.41 ± 1.98^d^	77.94 ± 1.28^b^	54.22 ± 2.49^e^	67.95 ± 1.43^b^
	120	77.76 ± 1.47^b^	88.30 ± 1.47^a^	59.67 ± 1.92d	72.44 ± 3.53^a^

Within the columns of a parameter, means expressed by a different letter are statistically significant (*p* < 0.05)

*TAC* Total Antioxidant Capacity

### DPPH free radical scavenging activity

DPPH free radical scavenging activity is one of the most significant assays for the assessment of antioxidant properties of food systems. In cheese, DPPH free radical scavenging activity indicates characteristics to the presence of peptides. DPPH Free radical scavenging activity of buffalo cheddar in conventional and accelerated ripening was greater than cow cheddar (Table 2). DPPH free radical scavenging activity of cow and buffalo went on increasing in conventional and accelerated ripening up to four months of ripening. DPPH free radical scavenging activity of 120 days old conventionally ripened cow and buffalo cheddar cheese was 44.56% and 63.27%. DPPH free radical scavenging activity of 120 days accelerated ripened cow and buffalo cheddar was 59.67 and 72.44%. DPPH free radical scavenging activity of 80 days rapid ripened old buffalo cheddar cheese was greater than 120 days rapid ripened cow cheddar. A study was performed to assess the antioxidant properties of peptides cheese ripening for six months, HPLC characterization revealed that antioxidant properties of cheese were strongly correlated with water-soluble peptides, DPPH free radicals scavenging activity of matured cheese was more than 95% [34]. Antioxidant characteristics of white brined cheese were investigated, antioxidant activity of water-soluble and fat-soluble fractions increased during the ripening [35].

### Fatty acid profile

Fatty acid profile of buffalo and cow cheddar cheese is presented in Table 3. Ripening temperature had a pronounced effect on fatty acid profile of buffalo and cow cheddar. After 120 days of conventional ripening, concentrations of short-chain fatty acids in buffalo and cow cheddar were 12.56% and 12.45% (*p* > 0.05). Accelerated ripening had a pronounced effect on fatty acid profile of buffalo cheddar cheese. After 120 days of accelerated ripening, concentration of short-chain fatty acids in buffalo and control cheddar were 14.36% and 13.86% (*p* < 0.05). After 120 days of conventional ripening, concentration of medium chain fatty acids in buffalo and cow cheddar was 43.97% and 45.71% (*p* > 0.05). After 120 days of Accelerated ripening, concentration of medium-chain fatty acids in buffalo and cow cheddar was 41.22% and 44.33% (*p* < 0.05). After 120 days of conventional ripening, concentrations of long-chain unsaturated fatty acids in buffalo and cow cheddar was 25.18% and 21.03% (*p* < 0.05). After 120 days of Accelerated ripening, concentration of long-chain unsaturated fatty acids in buffalo and cow cheddar was 18.62% and 16.92% (*p* < 0.05). In current investigation, changes in fatty acid profile of cheese was used an indication of degree of lipolysis. Cheese prepared from buffalo milk underwent relatively less lipolysis in conventional ripening and showed less transition in fatty acid profile as compared to cow cheddar. However, rapid ripening considerably enhanced the ripening of buffalo cheddar cheese. At the end of accelerated ripening of buffalo cheddar (120 days at 12 °C), concentrations of C_4:0_, C_6:0_, C_8:0_, C_10:0_, C_12:0_, C_14:0_, C_16:0_, C_18:0_, C_18:1_, C_18:2_ and C_18:3_ were 4.62%, 2.88%, 3.12%, 3.24%, 2.24%, 10.19%, 28.79%, 9.8%, 18.32%, 0.28% and .0.08%, respectively. At the end of accelerated ripening of cow cheddar cheese, concentrations of C_4:0_, C_6:0_, C_8:0_, C_10:0_, C_12:0_, C_14:0_, C_16:0_, C_18:0_, C_18:1_, C_18:2_ and C_18:3_ were 4.14%, 2.83%, 2.15%, 5.24%, 3.77%, 10.29%, 30.27%, 9.66%, 16.38%, 0.49% and 0.05%, respectively. For the estimation of degree of lipolysis in cheddar cheese in conventional and accelerated ripening, fatty acids profile was used. The reason for lower level of transition in fatty acid in conventional ripening may be attributed to the larger size of fat globules and lower moisture content in the curd. Michalski et al. [9] prepared camembert cheese from milk with large and small size fat globules; cheese underwent lower degree of lipolysis. Size of fat globules in buffalo milk is higher than cow milk; size of fat globules has pronounced effect on cheese ripening. In secondary and tertiary stages of lipolysis, fatty acids are metabolized to flavouring compounds [12]. Determination of fatty acid profile is an important characteristic to monitor the biochemical changes taking place in dairy products [36]. McSweeney and Sousa [26] used fatty acid profile as an important parameter to determine the degree of lipolysis in cheddar cheese.

**Table 3:** Changes in fatty acid composition of fresh and 120 days ripened cheese

Fatty Acids	4 °C				12 °C			
	Cow		Buffalo		Cow		Buffalo	
	Fresh	120 days old*	Fresh	120 days old*	Fresh	120 days old*	Fresh	120 days old*
C_4:0_	3.52 ± 0.11^e^	3.84 ± 0.19^c^	4.12 ± 0.01^b^	4.71 ± 0.21^a^	3.52 ± 0.11^d^	4.14 ± 0.08^b^	4.12 ± 0.01^b^	4.62 ± 0.22^a^
C_6:0_	2.32 ± 0.05^c^	2.57 ± 0.16^a^	2.39 ± 0.04^c^	2.61 ± 0.05^b^	2.32 ± 0.05^c^	2.83 ± 0.16^a^	2.39 ± 0.04^c^	2.88 ± 0.13^a^
C_8:0_	1.29 ± 0.09^e^	1.65 ± 0.09^d^	2.12 ± 0.04^c^	2.89 ± 0.07^b^	1.29 ± 0.09^e^	2.15 ± 0.22^c^	2.12 ± 0.04^c^	3.12 ± 0.18^a^
C_10:0_	3.18 ± 0.04^c^	4.39 ± 0.25^b^	1.81 ± 0.10^e^	2.35 ± 0.13^d^	3.18 ± 0.04^c^	5.24 ± 0.19^a^	1.81 ± 0.10^e^	3.24 ± 0.07^c^
C_12:0_	4.31 ± 0.13^a^	4.12 ± 0.07^b^	2.61 ± 0.06^d^	2.41 ± 0.09^e^	4.31 ± 0.13^a^	3.77 ± 0.08^c^	2.61 ± 0.06^d^	2.24 ± 0.09^f^
C_14:0_	11.95 ± 0.19^a^	10.42 ± 0.15^b^	11.82 ± 0.31^a^	11.26 ± 0.34^a^	11.95 ± 0.19^a^	10.29 ± 0.46	11.82 ± 0.31^a^	10.19 ± 0.53^b^
C_16:0_	32.42 ± 1.17^a^	31.17 ± 0.18^c^	31.67 ± 0.41^c^	30.27 ± 1.59^d^	32.42 ± 1.17^b^	30.27 ± 1.86^d^	31.67 ± 0.41^c^	28.79 ± 2.71^e^
C_18:0_	11.18 ± 0.71^a^	10.61 ± 0.46^b^	11.28 ± 0.52^a^	11.04 ± 0.76	11.18 ± 0.71^a^	9.66 ± 1.35^b^	11.28 ± 0.52^a^	9.80 ± 1.43^b^
C_18:1_	21.29 ± 0.82^b^	19.52 ± 1.19^e^	24.52 ± 0.35^a^	22.68 ± 1.28^c^	21.29 ± 0.82^d^	16.38 ± 0.69^g^	24.52 ± 0.35^a^	18.32 ± 0.88^f^
C_18:2_	1.76 ± 0.06^c^	1.34 ± 0.11^d^	2.65 ± 0.11^a^	2.21 ± 0.04^b^	1.762 ± 0.06^c^	0.49 ± 0.05^e^	2.65 ± 0.11^a^	0.28 ± 0.04^f^
C_18:3_	0.28 ± 0.04^b^	0.17 ± 0.02^c^	0.65 ± 0.04^a^	0.29 ± 0.03^b^	0.28 ± 0.04^b^	0.05 ± 0.01^e^	0.65 ± 0.04^a^	0.08 ± 0.02^d^

C_4_ to C_10_: Short-chain fatty acids

C_12_- C_16_: Medium-Chain Fatty Acids

C_18:0_ to C_18:3_: Long-Chain Fatty Acids

Within a row means denoted by a different letter are statistically significant (*p* < 0.05)

*: 120 days ripened

### Organic acids

**Fig. 1:**
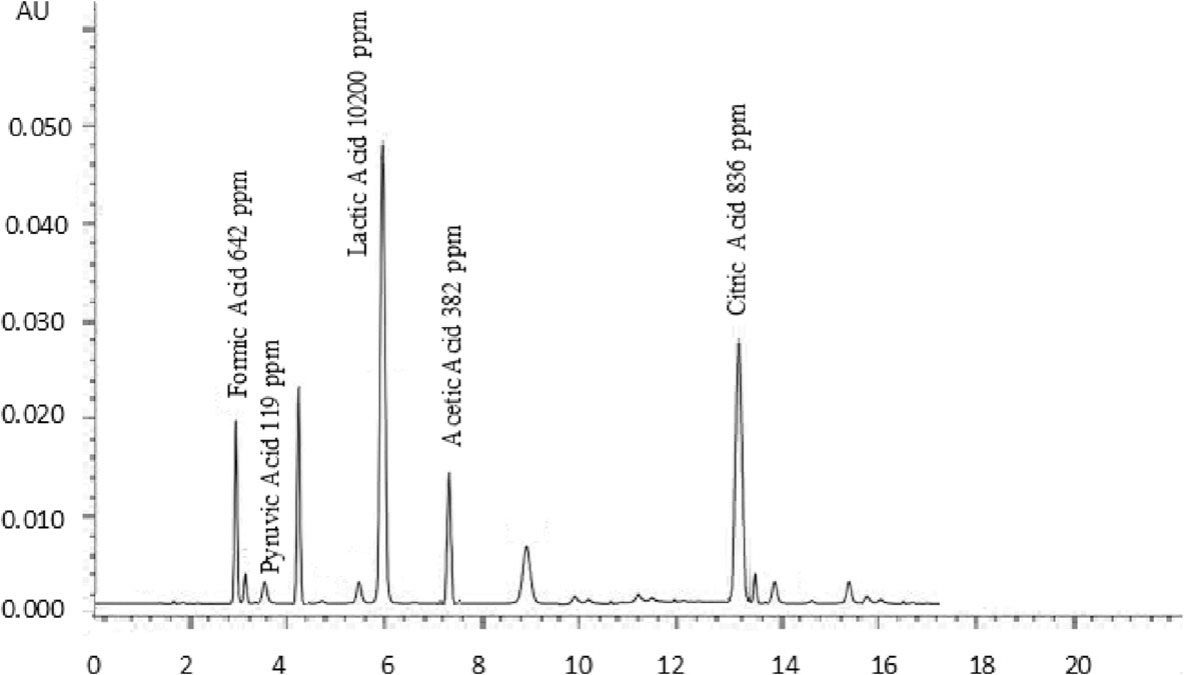
Effect of Conventional Ripening (4 C) on Concentration of organic acids in buffalo cheddar Cheese

**Fig. 2:**
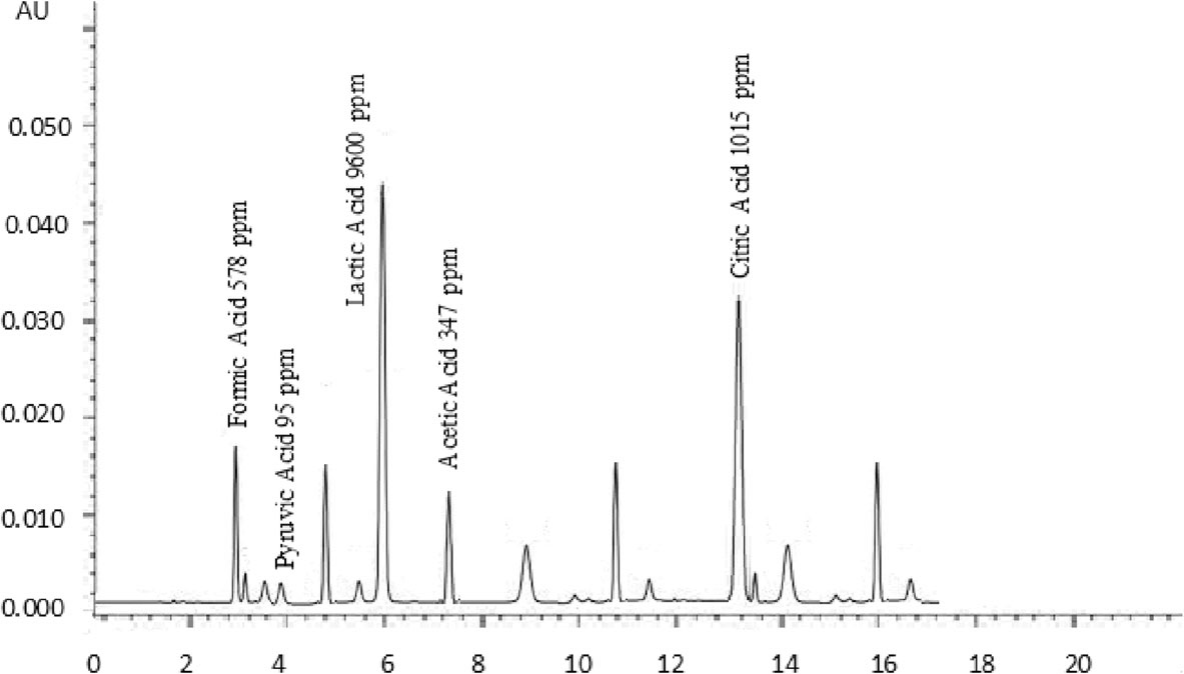
Effect of Conventional Ripening (4 °C) on Concentration of organic acids in cow cheddar Cheese

**Fig. 3:**
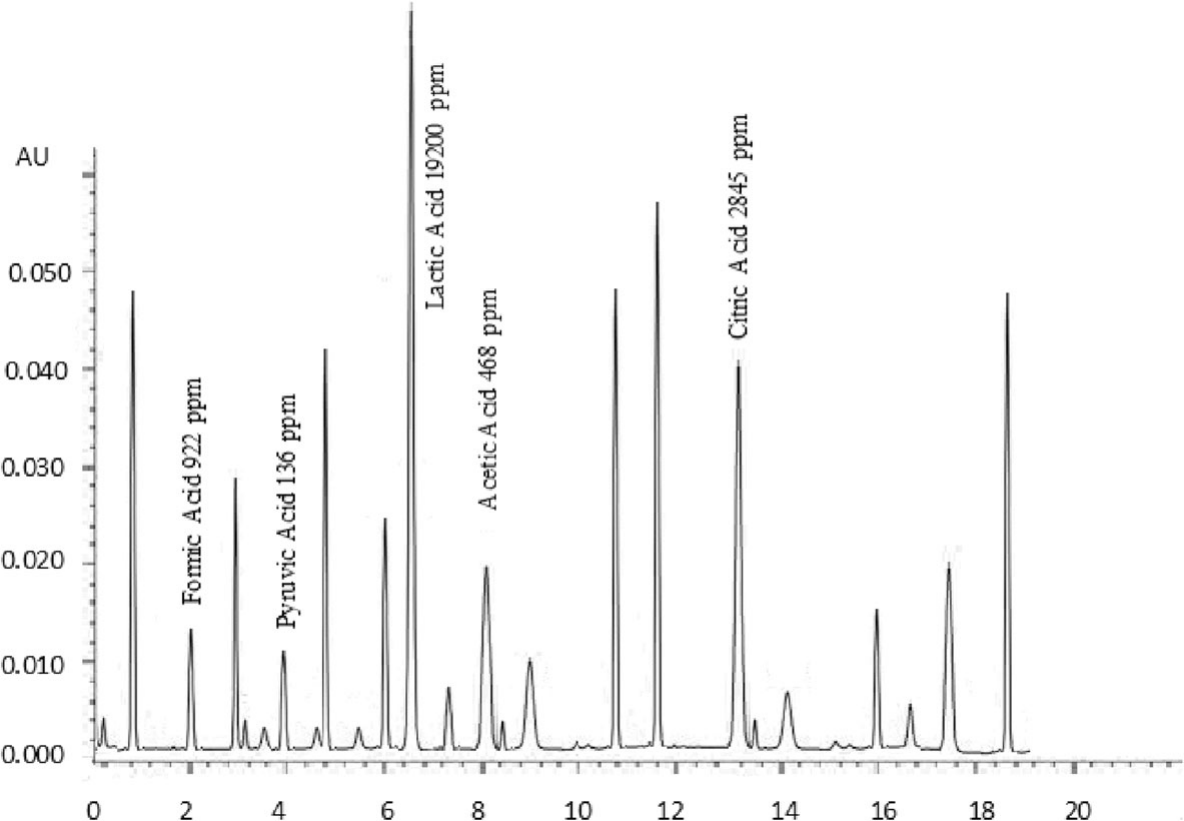
Fig. 2 Effect of Accelerated Ripening (12 °C) on Concentration of organic acids in Buffalo cheddar Cheese

**Fig. 4:**
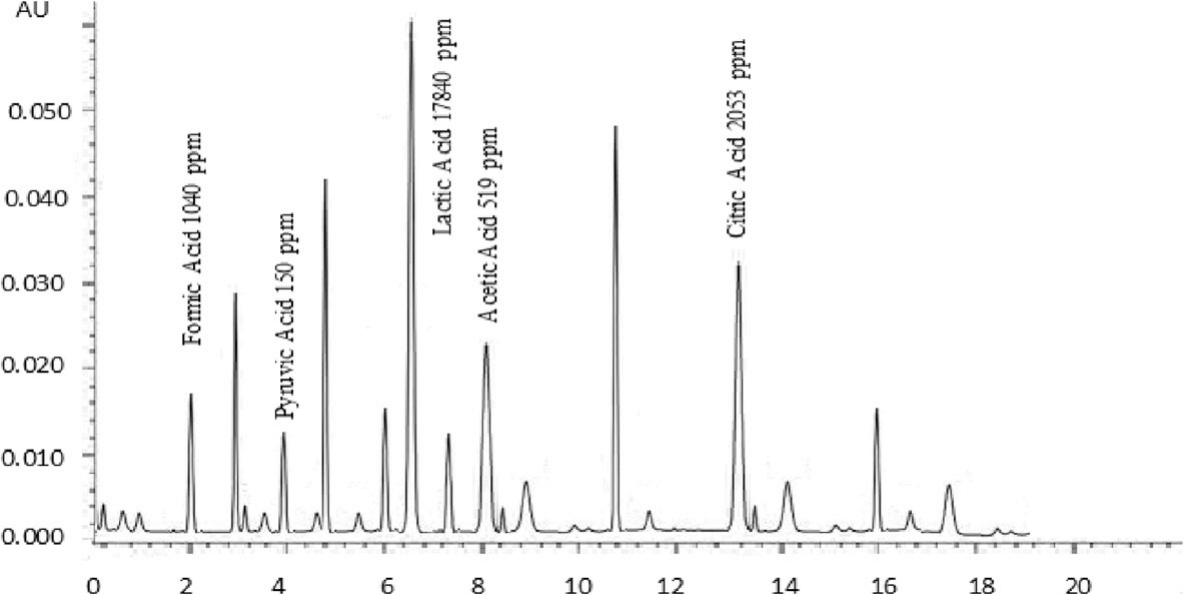
Effect of Accelerated Ripening (12 °C) on Concentration of organic acids in Cow cheddar Cheese

Ripening of cheese is a unique process in which biochemical and microbiological processes convert the chalky curd to a flavourful product; flavour perspectives of cheese depends upon several organic compounds such as lactones, methyl ketones, alcohols, phenolic substances and organic acids [37]. Organic acids and other flavouring compounds are produced during the ripening of cheese [38]. Results of organic acids of buffalo and cow cheddar in conventional and accelerated ripening are presented in Figs. 1, 2, 3 and 4. Concentrations of organic acids were substantially influenced by ripening temperatures. After 120 days of conventional ripening, the concentrations of formic, pyruvic, lactic, and acetic and citric acids in cow cheddar were 462, 119, 10,200, 382, 836 ppm, While the concentrations of the acids in buffalo cheddar ripened under the same conditions were 578, 95, 9600, 347 and 1015 ppm, respectively. Statistical analysis of the data showed that at the end of conventional ripening, amount of formic acid and citric acid in buffalo cheddar cheese was higher than cow milk, whereas, pyruvic, lactic and acetic acids were statistically higher in cow cheddar as compared to buffalo cheddar. After 120 days of accelerated ripening, amount of formic, pyruvic and citric acids in buffalo cheddar were statistically higher than cow cheddar cheese. After 120 days of accelerated ripening, concentrations of formic, pyruvic, lactic, and acetic and citric acids in cow cheddar were, 922, 136, 19,200, 468 and 2845 ppm, while concentrations of the acids in buffalo cheddar ripened under the same conditions were 1040, 150, 17,840, 519, 2053 ppm, respectively. Organic acids play an important role in the development of flavour of cheddar cheese. Flavour profile of Reggianito cheese was strongly correlated with the amount of organic acids [39]. Accelerated ripening had significant effect on the production of organic acids in buffalo cheddar cheese [25]. Accelerated ripening considerably shortened the ripening time for cheese [40]. Chemical characteristics of feta-type cheese prepared from buffalo and cow milk were comparable to each other [15].

### Lipolysis

Free fatty acids in samples buffalo and cow cheddar increased during the conventional and accelerated ripening, and all the testing intervals revealed an increasing trend (Table 4). Free fatty acid was influenced by the milk type and ripening temperature. After 120 days of conventional ripening, the free fatty acids in buffalo and cow cheddar were 0.22% and 0.26%, respectively (*p* < 0.05). Concentrations of free fatty acids in 120 days old rapid ripened buffalo and cow cheddar were 0.55% and 0.62%. Cow cheddar had a moisture content that could be appropriate justification for higher content of free fatty acids. During cheese ripening, indigenous and bacterial lipases hydrolyse the triglycerides [41]. Hydrolysis of triglycerides mainly depends upon moisture content, temperature, metal ion contamination and concentration of lipases. Catalytic role of temperature on the generation of free fatty acids in fats is scientifically established [42]. Free fatty acids of feta-type cheese prepared from buffalo milk were less than feta-type cheese prepared from cow milk [15]. Free fatty acids are produced during the primary stages of lipolysis, which are further degraded to flavouring compounds during the secondary and tertiary stages of cheese ripening [23]. Dairy products are discredited for having high amounts of dietary cholesterol which is regarded as one of the risk factor for cardiovascular disease. This has contributed to the avoidance of fat rich dairy products [43]. Concentration of cholesterol in cheddar cheese was significantly affected by milk type and ripening temperature. Cholesterol in cow and buffalo cheddar was 168 and 57 mg/100 g, respectively. After 120 days of conventional ripening, the concentration of cholesterol in cow and buffalo cheddar decreased from 168 mg/100 g to 88 mg/100 g and 57 mg/100 g to 25 mg/100 g, respectively. The decrease was 48% and 56% for the two kinds of cheddar cheese. In the case of accelerated ripening, the decrease in cholesterol concentration for the cow and buffalo cheddar cheese was 56% and 74%, respectively. Fermented dairy products have lower amount of dietary cholesterol than native milk. Reduction of cholesterol in fermented dairy products is due to the activities of cholesterol disrupting enzymes such as cholesterol reductase and cholesterol oxidase [44]. Kefir prepared from *Streptococci*, *lactobacilli*, yeast and acetic acid bacteria had lower levels of dietary cholesterol than parent milk, amount of cholesterol in kefir during 24 and 48 h storage period was 16% and 43% less than native milk [45]. Kefir culture decreased 84% cholesterol in the fermentation process and subsequent storage [46]. Folkertsma et al. [47] studied the effect of accelerated ripening on lipolysis in cheddar cheese which was exposed to 12 °C and 16 °C for a period of 9 months. The flavour score of cheese ripened at 16 °C was higher in the early stages. Ripening at 12 °C was recommended for the commercial/ large scale production of cheddar cheese. Buffalo cheddar has not been previously reported. Peroxide value measures the concentration of hydro peroxides formed in the first step of oxidation [36]. During conventional ripening (4 °C), cheese usually does not suffer from auto-oxidation because of the lower oxidation-reduction potential. After 120 days of conventional ripening, peroxide value of cow cheddar increased from 0.25 to 1.19 (MeqO_2_/kg) while that of buffalo cheddar increased from 0.23 to 1.08 (MeqO_2_/kg). After 120 days of accelerated ripening, the peroxide value of buffalo cheddar increased from 0.23 to 2.38 (MeqO_2_/kg) while that of cow cheddar increased from 0.25 to 2.59 (MeqO_2_/kg). After 120 days of accelerated ripening, peroxide value of cow and buffalo cheddar were 2.58 and 2.38(MeqO_2_/kg). The lower peroxide value in buffalo cheddar may be due to the presence of higher concentration of antioxidant substances [6]. Nadeem et al. [48] established a strong correlation between peroxide value and sensory score. Peroxide value of ripened cheese was higher than young cheese [49]. Iodine value measures the degree of unsaturation in fats and oils and depends upon the fatty acid profile of fat. Iodine value of buffalo and cow cheddar decreased during the conventional and accelerated ripening. During the process of ripening, the decrease in iodine value of cheddar cheese may be due to the saturation of some unsaturated sites by the oxygen. The decline in iodine value of cheddar cheese during the ripening period has been described in literature. Iodine value of ripened cheese was less than the fresh cheese [28].

**Table 4:** Lipolysis of cheddar cheese ripened at 4 °C and 12 °C

Ripening Temperature	Ripening Days	Cow Cheddar	Buffalo Cheddar	Cow Cheddar	Buffalo Cheddar	Cow Cheddar	Buffalo Cheddar	Cow Cheddar	Buffalo Cheddar
		FFA	FFA	PV	PV	IV	IV	Cholesterol	Cholesterol
		%	%	(MeqO_2_/kg)	(MeqO_2_/kg)	Cg/100 g	Cg/100 g	mg/100 g	mg/100 g
4 °C	0	0.11 ± 0.01^h^	0.12 ± 0.02^h^	0.25 ± 0.02^h^	0.23 ± 0.02^h^	36.7 ± 1.62^c^	39.4 ± 2.36^a^	168 ± 4.53^a^	57 ± 1.35^g^
	40	0.14 ± 0.02^h^	0.13 ± 0.01^h^	0.32 ± 0.06^h^	0.28 ± 0.04^h^	35.8 ± 1.37^e^	38.9 ± 3.15^b^	152 ± 3.65^b^	58 ± 1.42^h^
	80	0.19 ± 0.04^g^	0.18 ± 0.04^g^	0.78 ± 0.11^e^	0.65 ± 0.09^f^	34.5 ± 1.29^f^	37.4 ± 1.52^c^	129 ± 3.95^d^	38 ± 1.19^i^
	120	0.26 ± 0.03^e^	0.22 ± 0.05^f^	1.19 ± 0.08^c^	1.08 ± 0.15^d^	32.7 ± 0.81^g^	36.5 ± 1.89^d^	88 ± 2.39^f^	25 ± 1.10^j^
12 °C	0	0.11 ± 0.01	0.12 ± 0.02^h^	0.25 ± 0.02^h^	0.23 ± 0.02^h^	36.7 ± 1.62^d^	39.4 ± 2.36^a^	168 ± 4.53^a^	67 ± 1.35^g^
	40	0.27 ± 0.02^e^	0.19 ± 0.03^g^	0.43 ± 0.03	0.52 ± 0.05^g^	34.8 ± 0.95^f^	38.1 ± 4.34^b^	144 ± 2.33^c^	53 ± 3.22^h^
	80	0.47 ± 0.04^c^	0.39 ± 0.04^d^	1.26 ± 0.15^c^	1.18 ± 0.09^c^	32.4 ± 1.48^g^	35.6 ± 1.49^e^	107 ± 1.67^e^	31 ± 1.19^j^
	120	0.62 ± 0.03^a^	0.55 ± 0.02^b^	2.59 ± 0.08^a^	2.38 ± 0.16^b^	29.2 ± 1.34^h^	33.2 ± 2.74^g^	73 ± 3.42^g^	16 ± 0.56^k^

Within the columns of a parameter, means denoted by different letter are statistically different (*p* < 0.05)

*FFA* Free Fatty Acids (Oleic Acid)

*PV* Peroxide Value

*IV* Iodine Value (Wijs)

### Sensory evaluation

Results of sensory characteristics of cow and buffalo cheddar ripened in conventional and accelerated conditions are presented in Table 5. Ripening temperatures significantly affected the sensory score. Accelerated ripening significantly shortened the ripening time of buffalo cheddar cheese. Colour, flavour and texture score of rapid ripened 80 and 120 days old buffalo cheddar was not different from cow cheddar. These results evidenced that lack of flavour development in buffalo cheddar can be ameliorated by accelerated ripening for the better utilization of buffalo milk in cheese. Flavour score of cheddar cheese increased during the ripening period of 60 days [28].

**Table 5:** Sensory characteristics of 120 days old cheddar cheese ripened at 4 °C and 12 °C

Ripening Temperature	Ripening Days	Cow Cheddar	Buffalo Cheddar	Cow Cheddar	Buffalo Cheddar	Cow Cheddar	Buffalo Cheddar
		Color	Color	Flavor	Flavor	Texture	Texture
4 °C	40	6.8 ± 0.24^a^	6.4 ± 0.35^c^	6.5 ± 0.11^d^	6.2 ± 0.16^d^	6.2 ± 0.34^e^	6.0 ± 0.29^e^
	80	7.5 ± 0.15^a^	7.1 ± 0.19^b^	7.6 ± 0.19^b^	7.1 ± 0.10^c^	6.9 ± 0.16^c^	6.5 ± 0.13^d^
	120	8.1 ± 0.34^a^	7.6 ± 0.16^b^	8.0 ± 0.52^a^	7.5 ± 0.26^b^	8.1 ± 0.26^a^	6.8 ± 0.08^c^
12 °C	40	7.1 ± 0.14^a^	6.5 ± 0.42^c^	6.9 ± 0.15^c^	6.3 ± 0.12^d^	6.4 ± 0.15^d^	6.3 ± 0.12^e^
	80	8.0 ± 0.08^a^	8.1 ± 0.05^a^	8.2 ± 0.13^a^	7.7 ± 0.65^a^	8.0 ± 0.14^a^	7.9 ± 0.45^a^
	120	8.2 ± 0.17^a^	8.0 ± 0.11^a^	8.0 ± 0.16^a^	7.9 ± 0.53^a^	7.9 ± 0.31^a^	8.1 ± 0.27^a^

Within the columns of a parameter, means denoted by different letter are statistically different (*p* < 0.05)

## Conclusions

Flavour and texture score of buffalo cheddar ripened at 12 °C for 80 days was not significantly different from cow cheddar. These results suggest that accelerated ripening can be used to improve the sensory and chemical characteristics of buffalo cheddar cheese.
